# Impact of the COVID-19 pandemic on BMI: Its changes in relation to socio-demographic and physical activity patterns based on a short period

**DOI:** 10.1371/journal.pone.0266024

**Published:** 2022-03-24

**Authors:** Tahmina Akter, Zebunnesa Zeba, Ismail Hosen, Firoj Al-Mamun, Mohammed A. Mamun

**Affiliations:** 1 CHINTA Research Bangladesh, Dhaka, Bangladesh; 2 Department of Epidemiology, Bangladesh University of Health Sciences, Dhaka, Bangladesh; 3 Department of Public Health and Informatics, Jahangirnagar University, Dhaka, Bangladesh; 4 Department of Public Health, Daffodil International University, Dhaka, Bangladesh; University of Southern Queensland, AUSTRALIA

## Abstract

**Background:**

The COVID-19 pandemic is undoubtedly a major threat to the world. The preventive strategies designed to minimize the virus transmission by remaining at home, being isolated, and keeping social distance, which would substantially reform people’s lifestyle, physical activity, eating habits, etc. Consequently, those measures might create a disturbance in weight management and overweight. Therefore, how the COVID-19 pandemic has changed the physical activities of individuals and its impacts on the Body Mass Index (BMI) is explored herein.

**Methods:**

An online-based cross-sectional study collected data from 338 Bangladeshi adults in November 2020. The questionnaire included socio-demographics, health-related variables, physical activity-related variables, and diet measurement. Inferential statistics (i.e., chi-square test, McNemar test) were used to measure the associations between BMI and studied variables with a consideration of two scenarios (‘before’ and ‘during’ the pandemic inception), where *p*<0.05 was considered statistically significant.

**Results:**

Results showed that the prevalence of overweight was 30.5% ‘before’ the COVID-19 pandemic, which increased to 34.9% ‘during’ the pandemic; that means 4.4% of the participants significantly gained weight after the pandemic inception. There was no significant role of socio-demographic (e.g., gender, age, current residence, occupation) or physical activity-related factors (e.g., unavailability of outdoor space, not performing regular physical exercise, exercising with a partner) in changing the BMI status after the pandemic inception. However, following a proper diet plan during the COVID-19 pandemic was observed to decrease BMI status significantly.

**Conclusions:**

The present study suggests that a minor portion of the participants reported increasing their overweight status after the pandemic inception, whereas having a proper diet plan during the pandemic can significantly decrease BMI status. Therefore, the importance of the appropriate diet plan should be considered while implementing any policies.

## 1 Introduction

The COVID-19 is caused by a newly introduced virus named SARS-CoV-2, which has been turned into a pandemic. The first case of COVID-19 was detected in Wuhan, China, in late December 2019, and after its inception, the entire world has been infected with the virus very rapidly [1]. Because of its rapid transmission rate and disastrous effects, the WHO declared it as a pandemic in March 2020 [1]. However, in Bangladesh, where the present study was conducted, the first COVID-19 case was detected on 8 March 2020 [2], and the number of cases and deaths are being increased to date.

Like other countries, to control and suppress the transmission of this deadly infectious virus, the mandatory lockdown has been imposed by the government of Bangladesh, where all sorts of public activities are restricted [2,3]. The lockdown-related features include (i) social distancing for general people, (ii) quarantine for suspected cases, (iii) isolation for confirmed cases, etc. [4,5]. After these restrictions were imposed, people had to be confined, and their physical inactivity rate increased for an extended period. According to a recent study, the global trend in daily physical activity levels severely falls across different countries and age groups during confinement [6]. Therefore, people are gaining weight responsible for changes in their BMI status [7]. Physical inactivity also ultimately lessens organ system efficiency to tackle the virus infection and exacerbates the risk of destroying the immune, respiratory, cardiovascular, musculoskeletal systems, and brains [8,9]. At the same time, it has been said that stressful COVID-19 confinement situations disrupt daily life activities, which leads to unhealthy diet patterns and physical inactivity [9]. The unhealthy diet pattern and prolonged physical inactivity can alter BMI status and lead to a major health problem named the ‘obesity epidemic’, which is comorbid with multiple chronic severe illnesses [10].

Obesity is already a hidden pandemic in the twenty-first century [11]. Overweight is defined as a health impairment due to the body’s gradually aggregating unnecessary fats [12]. Adult people’s BMI more than or equal to 25 and more than or equal to 30 detects overweight and obesity, respectively [12]. The WHO estimated that the global prevalence of obesity had increased nearly 3-times from 1975 to 2016 [12]. In 2016, 39% of adults were reported as overweight; on the other hand, 13% were obese [12]. The recent estimation suggests an increment in incidence rate in Bangladesh (where the present study was carried out), that is, 17% overweight (including 3.3% obesity) was reported in the year 2013, which was 7% in 1980 [13,14]. In the context of the COVID-19 pandemic, obese people are more susceptible to the worse outcome of COVID-19 symptoms, including poor quality of life concerned with medical attention such as more extended hospital stay, type-2 respiratory failure, shift into ICU, and increased mortality rate [15–17].

As aforementioned, the COVID-19 crisis has already increased sedentary behaviors and unhealthy lifestyles, especially among youths [18–20], which are the common predictor that can impact BMI [21,22]. Some studies claimed that people are more reluctant to follow a healthy diet plan or regularly perform physical exercise [27], whereas overeating problems can increase BMI levels [23]. To prevent overweight and obesity, proper weight management, and regular physical exercise are considered crucial components, which can also help tackle cardiovascular health-related issues [24]. In this regard, studies investigating how the COVID-19 pandemic-related physical activity changes impact the BMI levels after its inception are needed for implementing strategies.

There is a limited study concerning the issues of overweight and its related factors in Bangladesh. For instance, a study investigating 450- Bangladeshi women reported 28% and 49% general and abdominal obesity rates, respectively [25]. Whereas urban married women had reported a prevalence of 34% overweight and obesity, the probability of being overweight and obese as increasing age and wealth index was also predicted [26]. Female adolescents were at a lower risk of being overweight than males in another study [27]. In the context of COVID-19, there is a study investigating the prevalence of physical inactivity and sedentary behaviors [28]. Obesity-related issues in COVID-19 morbidity and mortality can play a critical role by lowering immunity, which is also predicted for the Bangladeshis [29]. However, none of those above studies provided information on (i) the prevalence of overweight/obesity for ‘before’ and ‘during’ the pandemic, (ii) the associations of socio-demographics and physical activities with the BMI changes ‘during’ the pandemic (compared to ‘before’ the pandemic). Therefore, the present study’ objectives are to investigate aforementioned knowledge gap for the first time in Bangladesh.

## 2 Methods

### 2.1 Study procedure, participants, and ethics

An online-based cross-sectional descriptive study was conducted among Bangladeshi adults utilizing a convenience sampling technique in November 2020. A structured questionnaire concerning socio-demographic, health-related issues, physical activity, and diet plan was adhered to a survey link to collect data. Later on, the survey link was circulated through utilizing different popular social media platforms (e.g., Facebook, WhatsApp, Imo, etc.). Participation was voluntary in this survey, and before data collection, informed consent was taken from the participants after describing the aims and purpose of the study. Besides, the anonymity and confidentiality of their data were also ensured, along with the right to withdraw their participation whenever they wanted. In this study, the participant’s inclusion criteria were being Bangladeshi people and age ≥18 years. This study adhered to the Helsinki Declaration 2013 guideline [30]. Also, before the project’s initiation, formal ethical approval was obtained from the ethics committee at the Institute of Allergy and Clinical Immunology of Bangladesh (reference number: IRBIACIB/CEC/03202028/653). Initially, about 342 online survey responses were recorded, and after removing the incomplete responses, 338 participants were included for final analysis.

### 2.2 Measures

#### 2.2.1 Socio-demographic factors

A structured questionnaire was used for collecting the participants’ information about gender, age, current residence (e.g., village, Upazila [sub-district], or district [city]), types of the house (e.g., lower = separate pucca house, middle = tin shed house, or high quality = flat house/apartment building) and occupation (e.g., students, service holder, health care professionals, businessman, engineer, housewife, and others).

#### 2.2.2 Health-related variables

Participants were asked questions regarding their health-related issues based on ‘before’ and ‘after’ 6-month of the pandemic inception at the same period of time-based on their memory recall. Suffering from migraine pain was assessed using 3- point Likert item (i.e., yes, sometimes, and never) as a previous study indicated that migraine sufferers were more likely to be overweight, obese, or morbidly obese, and found a significant relationship between BMI and migraine attack frequency [31]. Self-rated height and weight were collected to measure the BMI (body mass index), where BMI categorization was followed by the WHO guideline [12]. For calculating the BMI changes, the assessed BMI status during the pandemic was subtracted with the status of the non-pandemic to observe whether the status was increased, decreased, or became neutral during the COVID-19 period. For example, if someone had a BMI of 27.5 kg/m^2^ before the pandemic, and it was 25.5 kg/m^2^ during the pandemic, then they were assigned to be decreased BMI status.

#### 2.2.3 Physical activity-related variables

Participants were asked questions regarding their physical activities based on two time periods (that is, ‘before’ and ‘after’ 6-month of the pandemic inception). Dichotomous responses (i.e., yes or no) were collected for the questions such as the presence of outdoor space close to the house for performing physical exercise and performing physical exercise regularly. Also, the participants’ physical exercise performing pattern (i.e., never, alone dual, group) was assessed [32].

#### 2.2.4 Diet plan during the pandemic

Participants’ diet plan during the pandemic was also assessed for this study. For this purpose, a dichotomous response (yes/no) was used to assess if the participants were following any diet plan during the COVID-19 pandemic.

### 2.3 Statistical analysis

Data were analyzed using the IBM Statistical Package for Social Science (SPSS) version 22 and Microsoft excel 2019. Before performing the formal analysis, data were obtained from the *Google form* and cleaned using Microsoft Excel 2019. Descriptive (i.e., percentages and frequencies) and inferential statistics (i.e., Chi-square test, McNemar test) were performed by the IBM SPSS. BMI status during ‘before’ and ‘during’ the pandemic and its changes after the pandemic inception were considered as the dependent variables, whereas socio-demographics, and physical activities-related variables were the predictors. In Tables 1 & 2, based on Chi-square tests, the associations of socio-demographics (Table 1) and physical activity-related variables (Table 2) with both ‘before’ and ‘during’ the COVID-19 pandemic were presented. In addition, where the relationships between changes in BMI (increased, decreased, and neutral) during the pandemic from before the pandemic and the studied socio-demographic and physical activity -related variables were presented based on the Chi-square tests (Tables 3 & 4). The difference between ‘before’ the COVID-19 pandemic BMI and ‘during’ the COVID-19 pandemic BMI was assessed using the McNemar test. In the present study, *p*<0.05 was considered statistically significant.

**Table 1:** Associations between socio-demographic variables and BMI status.

Variables	Total (n, %)	Before the COVID-19 pandemic			During the COVID-19 pandemic		
		Underweight (n, %)	Normal (n, %)	Overweight (n, %)	Underweight (n, %)	Normal (n, %)	Overweight (n, %)
**Gender**							
Female	123 (36.4)	11 (8.9)	77 (62.6)	35 (28.5)	12 (9.8)	73 (59.3)	38 (30.9)
Male	215 (63.6)	18 (8.4)	129 (60.0)	68 (31.6)	16 (7.4)	119 (55.3)	80 (37.2)
**χ**^**2**^ **/ *p* value**		.375/.829			1.620/.445		
**Age group**							
<23 years	127 (37.6)	21 (16.5)	82 (64.6)	24 (18.9)	19 (15)	77 (60.6)	31 (24.4)
23–30 years	122 (36.1)	4 (3.3)	77 (63.1)	41 (33.6)	4 (3.3)	67 (54.9)	51 (41.8)
>30 years	89 (26.3)	4 (4.5)	47 (52.8)	38 (42.7)	5 (5.6)	48 (53.9)	36 (40.4)
**χ**^**2**^ **/ *p* value**		26.771/.000			18.274/.001		
**Current residence**							
Village	60 (17.8)	9 (15)	37 (61.7)	14 (23.3)	5 (8.3)	40 (66.7)	15 (25.0)
Upazilla	106 (31.4)	6 (5.7)	68 (64.2)	32 (30.2)	9 (8.5)	63 (59.4)	34 (32.1)
District	172 (50.9)	14 (8.1)	101 (58.7)	57 (33.1)	14 (8.1)	89 (51.7)	69 (40.1)
**χ**^**2**^ **/ *p* value**		5.706/.222			5.209/.267		
**House type**							
Lower	59 (17.5)	11 (18.6)	36 (61.0)	12 (20.3)	8 (13.6)	37 (62.7)	14 (23.7)
Middle	69 (20.4)	4 (5.8)	46 (66.7)	19 (27.5)	4 (5.8)	44 (63.8)	21 (30.4)
High	210 (62.1)	14 (6.7)	124 (59.0)	72 (34.3)	16 (7.6)	111 (52.9)	83 (39.5)
**χ**^**2**^ **/ *p* value**		12.164/.016			7.926/.094		
**Occupation**							
Student	176 (52.1)	24 (13.6)	114 (64.8)	38 (21.6)	20 (11.4)	109 (61.9)	47 (26.7)
Service holder	79 (23.4)	1 (1.3)	45 (57)	33 (41.8)	3 (3.8)	41 (51.9)	35 (44.3)
HCPs	24 (7.1)	1 (4.2)	17 (70.8)	6 (25.0)	1 (4.2)	14 (58.3)	9 (37.5)
Businessman	11 (3.3)	2 (18.2)	5 (45.5)	4 (36.4)	2 (18.2)	5 (45.5)	4 (36.4)
Others	14 (4.1)	0 (0)	7 (50)	7 (50)	0 (0)	6 (42.9)	8 (57.1)
Engineer	19 (5.6)	0 (0)	10 (52.6)	9 (47.4)	1 (5.3)	10 (52.6)	8 (42.1)
Housewife	15 (4.4)	1 (6.7)	8 (53.3)	6 (40)	1 (6.7)	7 (46.7)	7 (46.7)
**χ**^**2**^ **/ *p* value**		29.078/.004			17.662/.126		

**Table 2:** Associations between physical activities -related variables and BMI status.

	Before the COVID-19 pandemic				During the COVID-19 pandemic			
	Total (n, %)	Underweight (n, %)	Normal (n, %)	Overweight (n, %)	Total (n, %)	Underweight (n, %)	Normal (n, %)	Overweight (n, %)
**Suffering from migraine pain**								
Yes	97 (28.70)	11 (11.3)	54 (55.7)	32 (33.0)	124 (36.7)	15 (12.1)	70 (56.5)	39 (31.5)
Sometimes	80 (23.7)	6 (7.5)	51 (63.7)	23 (28.7)	64 (18.9)	4 (6.3)	40 (62.5)	20 (31.3)
Never	161 (47.6)	12 (7.5)	101 (62.7)	48 (29.8)	150 (44.4)	9 (6)	82 (54.7)	59 (39.3)
**χ**^**2**^ **/ *p-* value**	-	2.141/.710			-	5.440/.245		
**Maintaining diet plan**								
No	-	-	-	-	249 (73.7)	22 (8.8)	141 (56.6)	86 (34.5)
Yes	-	-	-	-	55 (16.3)	3 (5.5)	29 (52.7)	23 (41.8)
**χ**^**2**^ **/ *p* -value**	-	-			-	1.414/.493		
**Having outdoor space**								
No	121 (35.8)	9 (7.4)	80 (66.1)	32 (26.4)	124 (36.7)	9 (7.3)	74 (59.7)	41 (33.1)
Yes	217 (64.2)	20 (9.2)	126 (58.1)	71 (32.7)	214 (63.3)	19 (8.9)	118 (55.1)	77 (36.0)
**χ**^**2**^ **/ *p*-value**	-	2.116/.347			-	.725/.696		
**Performing regular physical exercise**								
No	173 (51.2)	16 (9.2)	101 (58.4)	56 (32.4)	221 (65.4)	21 (9.5)	117 (52.9)	83 (37.6)
Yes	165 (48.8)	13 (7.9)	105 (63.6)	47 (28.5)	117 (34.6)	7 (6)	75 (64.1)	35 (29.9)
**χ**^**2**^ **/ *p*-value**	-	.986/.611			-	4.101/.129		
**Exercise performing pattern**								
Never	140 (41.4)	11 (7.9)	83 (59.3)	46 (32.9)	164 (48.5)	16 (9.8)	89 (54.3)	59 (36.0)
Alone	136 (40.2)	13 (9.6)	88 (64.7)	35 (25.7)	137 (40.5)	10 (7.3)	81 (59.1)	46 (33.6)
Dual	18 (5.3)	0 (0)	10 (55.6)	8 (44.4)	13 (3.8)	0 (0)	7 (53.8)	6 (46.2)
Group	44 (13.0)	5 (11.4)	25 (56.8)	14 (31.8)	24 (7.1)	2 (8.3)	15 (62.5)	7 (29.2)
**χ**^**2**^ **/ *p*-value**	-	5.209/.517			-	2.960/.814		

**Table 3:** Associations between socio-demographic variables and BMI change.

	BMI changes during the pandemic				
	Increased (n, %)	Decreased (n, %)	Neutral (n, %)	χ^2^ test value	*p-*value
**Gender**					
Female	7 (5.7)	5 (4.1)	111 (90.2)	3.541	.170
Male	25 (11.6)	11 (5.1)	179 (83.3)		
**Age group**					
<23 years	11 (8.7)	2 (1.6)	114 (89.8)	10.457	.033
23–30 years	17 (13.9)	7 (5.7)	98 (80.3)		
>30 years	4 (4.5)	7 (7.9)	78 (87.6)		
**Current residence**					
Village	6 (10.0)	1 (1.7)	53 (88.3)	4.644	.326
Upazilla	6 (5.7)	7 (6.6)	93 (87.7)		
District	20 (11.6)	8 (4.7)	144 (83.7)		
**House type**					
Lower	6 (10.2)	1 (1.7)	52 (88.1)	1.549	.818
Middle	6 (8.7)	4 (5.8)	59 (85.5)		
High	20 (9.5)	11 (5.2)	179 (85.2)		
**Occupation**					
Student	18 (10.2)	5 (2.8)	153 (86.9)	13.306	.347
Service Holder	6 (7.6)	6 (7.6)	67 (84.8)		
HCPs	4 (16.7)	1 (4.2)	19 (79.2)		
Businessman	0 (0)	0 (0)	11 (100)		
Others	2 (14.3)	1 (7.1)	11 (78.6)		
Engineer	1 (5.3)	3 (15.8)	15 (78.9)		
Housewife	1 (6.7)	0 (0)	14 (93.3)		

**Table 4:** Associations between physical activities -related variables and BMI change.

	BMI changes during the pandemic				
	Increased (n, %)	Decreased (n, %)	Neutral (n, %)	χ^2^ test value	*p*-value
**Before the pandemic—Suffering from migraine pain**					
Yes	9 (9.3)	7 (7.2)	81 (83.5)	2.446	.654
Sometimes	6 (7.5)	3 (3.8)	71 (88.8)		
Never	17 (10.6)	6 (3.7)	138 (85.7)		
**During the pandemic—Suffering from migraine pain**					
Yes	11 (8.9)	7 (5.6)	106 (85.5)	.688	.953
Sometimes	6 (9.4)	2 (3.1)	56 (87.5)		
Never	15 (10.0)	7 (4.7)	128 (85.3)		
**Before the pandemic—Having outdoor space**					
No	14 (11.6)	7 (5.8)	100 (82.6)	1.539	.463
Yes	18 (8.3)	9 (4.1)	190 (87.6)		
**During the pandemic—Having outdoor space**					
No	13 (10.5)	9 (7.3)	102 (82.3)	3.136	.208
Yes	19 (8.9)	7 (3.3)	188 (87.9)		
**Before the pandemic—Performing regular physical exercise**					
No	13 (7.5)	6 (3.5)	154 (89.0)	3.055	.217
Yes	19 (11.5)	10 (6.1)	136 (82.4)		
**During the pandemic—Performing regular physical exercise**					
No	21 (9.5)	7 (3.2)	193 (87.3)	3.484	.175
Yes	11 (9.4)	9 (7.7)	97 (82.9)		
**Before the pandemic–Exercise performing pattern**					
Never	10 (7.1)	5 (3.6)	125 (89.3)	3.835	.699
Alone	17 (12.5)	8 (5.9)	111 (81.6)		
Dual	2 (11.1)	1 (5.6)	15 (83.3)		
Group	3 (6.8)	2 (4.5)	39 (88.6)		
**During the pandemic–Exercise performing pattern**					
Never	14 (8.5)	4 (2.4)	146 (89.0)	7.382	.287
Alone	14 (10.2)	8 (5.8)	115 (83.9)		
Dual	2 (15.4)	2 (15.4)	9 (69.2)		
Group	2 (8.3)	2 (8.3)	20 (83.3)		
**During the pandemic–Maintaining diet plan**					
No	27 (10.8)	9 (3.6)	213 (85.5)	9.580	.008
Yes	2 (3.6)	7 (12.7)	46 (83.6)		

## 3 Results

### 3.1 Characteristics of the participants

Of the total sample (N = 338), the majority of the participants were male (63.6%) and young adults (37.6%). The mean age of the participants was 27.07 ± 8.05 years (age range was 18–65). Over half of them were students (52.1%), and the majority portion lived in a high-quality house (62.1%) and district area (50.9%) (**Table 1**). About 28.7% of the participants reported experiencing migraine pain ‘before’ the COVID-19 pandemic, whereas 36.7% ‘during’ the pandemic. Nearly three-fourths of the participants (i.e., 73.7%) had no healthy diet plan ‘during the pandemic. Similarly, about 64.2% reported having outdoor space for exercise ‘before’ the pandemic, whereas a negligible reduction (63.3%) was found ‘during’ the pandemic. Nearly half (48.8%) of the participants reported performing regular physical exercise ‘before’ the pandemic, but it was 34.6% for ‘during’ the pandemic (**Table 2**).

### 3.2 Prevalence of the overweight, underweight, normal, and obesity

**Fig 1:**
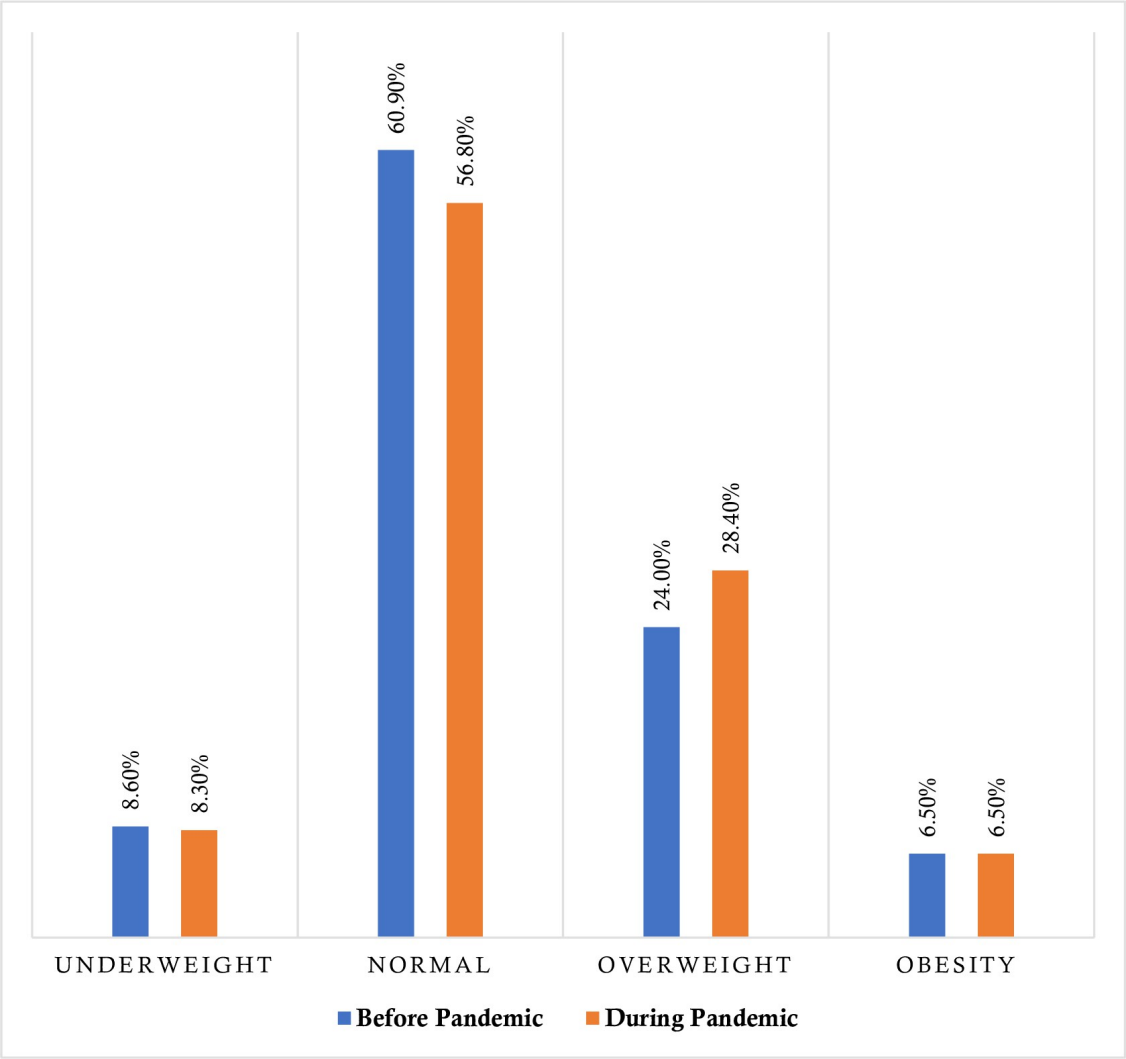
Prevalence of BMI status ‘before’ and ‘after’ 6-month of the pandemic inception.

The prevalence rate of overweight, underweight, normal and obesity is reported in **Fig 1**. About 30.5% of the participants reported being overweight (including obese) ‘before’ the COVID-19 pandemic, whereas it was 34.9% for ‘during’ the COVID-19 pandemic. That means the prevalence of overweight increased by 4.4% after the pandemic inception (*p =* 0.046). In addition, normal and underweight status was decreased with a rate of 4.1% and 0.3%, respectively, where the rate remains stable for obesity (**Fig 1**).

### 3.3 Factors associated with BMI Status

The gender was not significantly associated with BMI status of either ‘before’ or ‘during’ the COVID-19 pandemic. On the other hand, the age group was significantly associated with both ‘before’ and ‘during’ pandemic BMI statuses (*χ*^2^ = 26.771, *p<*0.001, and *χ*^2^ = 18.274, *p*<0.05, respectively). Where, among young adults (23–30 years) overweight was 33.6%, which increased to 41.8% after the inception of the pandemic. Participant’s occupation was significantly associated with only ‘before’ pandemic BMI status (*χ*^2^ = 29.078, *p*<0.05). Where 21.6% to 26.7% overweight rate increased among students after the inception of the pandemic (**Table 1**). In addition, none of the physical activities-related variables was significantly associated with the status of BMI of either ‘before’ or ‘during’ the pandemic (**Table 2**).

### 3.4 Factors associated with BMI changes

The associations of the BMI changes after the pandemic inception with socio-demographic and physical activities-related variables are presented in **Tables 3 and 4**. Unfortunately, none of the socio-demographics was found significantly associated with the BMI changes, except the age group (*χ*^2^ = 10.457, *p*<0.05) (**Table 3**). In addition, having a diet plan during the pandemic was significantly associated with the BMI change (*χ*^2^ = 9.580, *p<*0.05). That is, 10.8% of the participants without having any diet plan reported increasing their BMI after the pandemic inception; whereas, 12.7% reported decreasing their BMI by following a proper diet plan. Furthermore, suffering from migraine pain was not significantly associated with altering BMI status in the present study. (**Table 4**).

## 4 Discussion

With disrupting people’s quality of life, the COVID-19 pandemic has adversely impacted physical and mental health worldwide [4,5]. Individuals suffering from chronic health problems are highly susceptible to develop severe complications from this disease, which is also alleged to increase the mortality rate [33,34]. The virus has a wide range of signs and symptoms, from asymptomatic to mild and severe clinical symptoms like severe respiratory failure in the body [35,36]. Some of the studies affirmed that maintaining a healthy lifestyle and performing regular physical activities can help to minimize severe complications of COVID-19 infection [37,38]. Besides, mental health problems like anxiety, depression, sleep problems, even suicidality, etc., are associated with less physical activity [32,39–42], which is also observed in Bangladesh as reported in the recent systematic reviews [43,44]. Given the importance of physical activities in maintaining physical and mental health, this study can add value in the context of the COVID-19 pandemic.

At the earlier period of the COVID-19 pandemic, several public health preventive strategies were adopted to suppress the virus transmission. Unfortunately, these measures have shown adverse effects. Due to social movement restrictions, people could not go outside, which persuaded people to be physically inactive. As a result, they are gaining weight mostly, which impacts their BMI. The present study finds a 4.4% increment of the overweight (indicates BMI changes) prevalence after the pandemic inception (30.5% and 34.9%; ‘before’ and ‘after’ 6-month of the pandemic inception). However, the rate of overweight during the pandemic (34.9%) is remarkably similar to the previous studies [45,46], where 38.8% and 37.3% of the participants increased their body weight on an average of 2.6 kg between 1 to 3 kg, respectively. In contrast with overweight situations, the prevalence of underweight (0.3%) and normal (4.1%) status have lessened during the COVID-19 pandemic. The reason is due to increased body weight resulting in BMI changes. In line with the situation, the confinement effect on eating habits also leads to people increasing their BMI status; that is, 48.6% overweight is reported in Italy [47]. Another study reported that 43% reduced physical activity, where 34% of participants had overeaten [48].

Obesity is a modifiable risk factor of non-communicable diseases as well as the COVID-19. Obese patients were more vulnerable to developing severe complications of the COVID-19 disease [15–17]; which is also reported in the previous viral outbreaks [49]. Previous studies reported that lockdown-related factors (i.e., mandatory lockdown, self-isolation, and social movement limitation) lead to continuous stress and panic in human life [44], which ultimately alter our eating habits and lead us to an unhealthy diet which is a major contributing factor for developing obesity [50–52]. Therefore, a proper diet plan can reduce overweight, which is also reflected in this study. That is, 12.7% of individuals reported reducing their weight by following an appropriate diet plan during the COVID-19 pandemic. It is said that participants with higher BMI negatively change in eating and physical activity behavior and obstacles to manage weight during the lockdown period [23]. Another study observed that about 56% of participants had snacks more frequently, and overeating and a low-quality diet with lower physical activity levels exacerbate the problem [23]. Also, 28% of participants had pro-healthy diet patterns, and 19% had an unhealthy diet pattern during the lockdown period, which is common among adults (age more than 40 years), having children, and unemployed people [48].

Adequate physical activities result in a positive impact on the immune system. In addition, chronic and low-grade inflammation, commonly found in central obesity is preventable by regular physical activity, consequently reduce the risk of chronic conditions like diabetes mellitus, cardiovascular diseases, etc. [53]. Physical activity can play a role in reverse this grave situation, as it is inversely associated with being overweight. Therefore, physical activity is a significant lifestyle behavior in long-term weight reduction, and maintaining an ideal weight, and there is no beyond following it [54]. The effectiveness of physical activity can also be increased by dietary modification [54]. By increasing total energy expenditure, physical activity can allow to maintain balanced energy or lose weight and slow down the growth of abdominal obesity. Hill et al. [55] stated that to keep sustainable weight loss, 60–90 min/day moderate frequency of physical activity is needed. In addition, to keep the energy balanced and daily energy expenditure, voluntary activities can play a role [56]; whereas regular physical activity can help in controlling overeating behavior and reducing anxiety-related weight gain [57]. However, Balanzá–Martínez et al. [58] advised that to prevent the future burden of psychiatric and cardiometabolic disorders after the COVID-19 era, strategies focusing on healthiest lifestyle behavior, where a healthy diet plan and regular physical exercise at home should be included. The WHO [59] started a campaign “Be Active” during the COVID-19 pandemic to motivate people to be physically active. Walking or stretching for 3–4 minutes daily and following a healthy diet to boost the immune system is highly recommended [59]. Regular physical activity and a healthy diet can help prevent, fight and recover from this disease and prevent future obesity-related complications.

Being a cross-sectional study with small sample size, the present study can be limited. Also, the sample was not representative and was collected by the online sampling method; thus, the results cannot be generalized. In addition, the study primarily relied on self-reported questionnaires (there is a possibility of memory recall bias) to assess the outcomes and failed to establish a clinical diagnosis, which may limit the study. Thus, further studies involving more robust methodologies with a larger sample size are recommended to determine the actual reason for being overweight and obese and establish causal inference.

However, being an exploratory study, the findings reported herein will be helpful for future studies and health policymakers to adopt strategies preventing the double burden of diseases, COVID-19 and obesity. For example, increasing BMI due to lockdown might increase the vulnerability and challenges of being obese for case management; therefore, it may raise the cost of healthcare expenditure. Furthermore, it is alleged that there is a lack of proper planning, implementation, and monitoring, although the government introduced many NCD-related policies or programs [60]. But given the unpredictable nature of the pandemic, health issues related to the pandemic (e.g., lockdown increasing the risk of obesity) should be given in the priority list with the effectiveness of national policies that are driven and implemented by the experts, where this study finding can be used.

## 5 Conclusion

In conclusion, the present study has revealed that the BMI status during the COVID-19 pandemic has been increased by waxing the overweight rate. Besides, maintaining a proper diet plan was the only factor that significantly altered BMI status during this pandemic. Therefore, although minor portions of the people reported increasing their BMI status, concerns should be provided in regulating the modifiable risk factors. Therefore, developing reliable and fitted recommendations and tools to promote health and avoid the future burden of non-communicable diseases associated with obesity is highly recommended with a special concern to the risky individuals.

## Supporting information

S1 Dataset (SAV)
